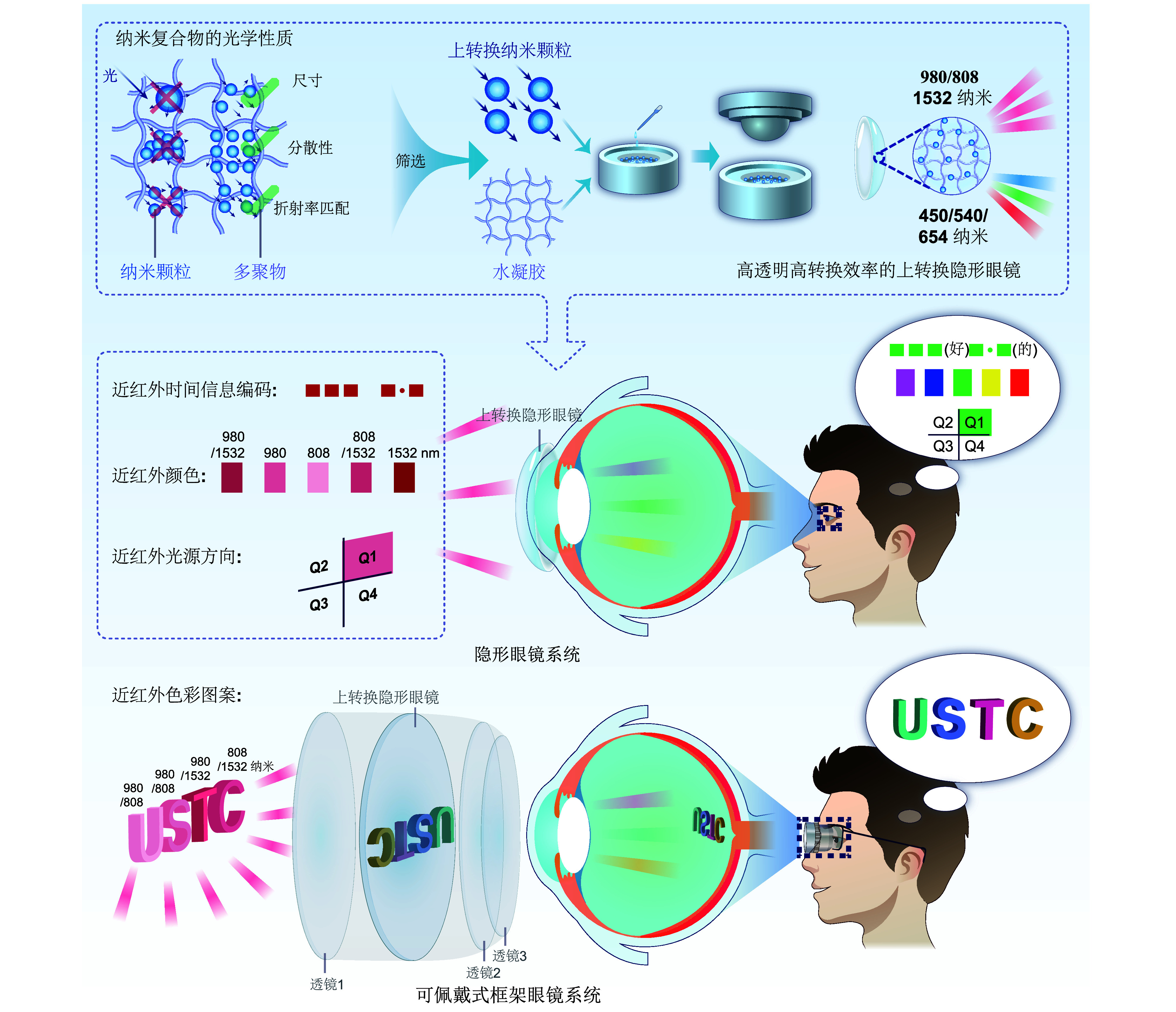# Two breakthroughs recommended by BSC recognized as Top 10 Scientific Advances in Life Sciences of China 2025

**DOI:** 10.52601/bpr.2025.250903

**Published:** 2026-02-28

On December 9^th^, 2025, the China Union of Life Science Societies announced the "Top 10 Scientific Advances in Life Sciences of China 2025." Among the prestigious selections were two groundbreaking projects recommended by the Biophysical Society of China (BSC): "Regulation and new strategies for host homeostasis and imbalance by novel microbial metabolites" led by Jiang Changtao, and "Near-infrared spatio-temporal color vision via upconversion contact lenses" led by Xue Tian.

The annual selection by the China Union of Life Science Societies aims to highlight significant achievements in life sciences, promoting research and technological innovation. Since its inception in 2015, this initiative has showcased major scientific breakthroughs in China over 11 consecutive years.

These recognitions underscore China's leading role in life sciences, setting the stage for future innovations in biophysical research and technology.

## Regulation and new strategies for host homeostasis and imbalance by novel microbial metabolites

The project led by Jiang Changtao and his team from Peking University focuses on the diversity and dynamic transformation of metabolites, crucial for maintaining metabolic homeostasis and understanding disease progression. The team developed an AI-based system to identify novel gut microbial metabolites, discovering new compounds like bile acid double-tailin and tryptophan bile acids. They identified orphan receptor MRGPRE as a new target for metabolic diseases and discovered new secondary metabolites from gut symbiotic fungi, which improve metabolic diseases by regulating host sphingolipids. This work opens new avenues for targeted interventions.

**Figure 1:**
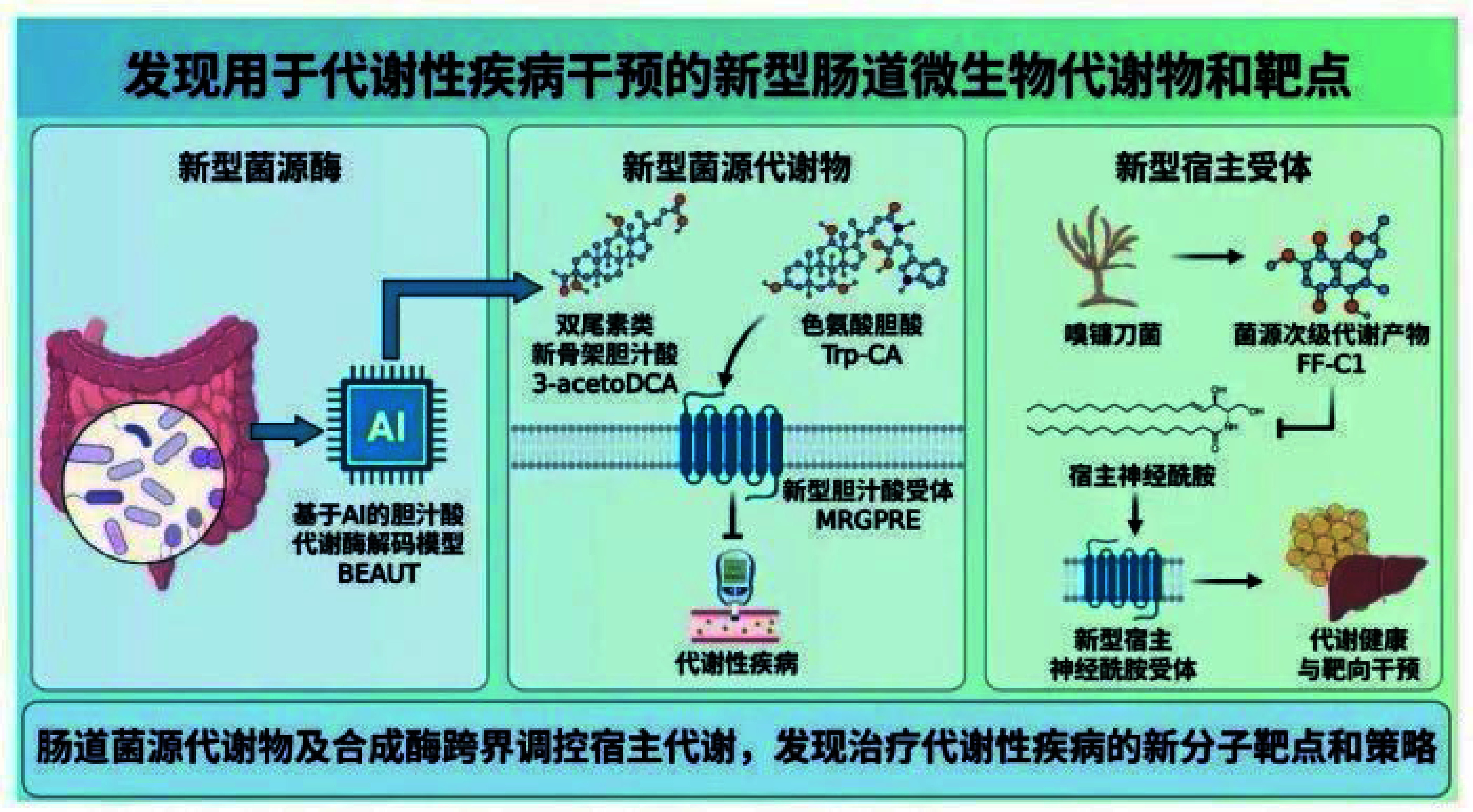


## Near-infrared spatio-temporal color vision via upconversion contact lenses

Xue Tian's team from the University of Science and Technology of China, in collaboration with teams from Fudan University, developed innovative contact lenses that integrate nanomaterials capable of converting infrared light to visible light. These lenses allow humans to perceive near-infrared light in multiple dimensions (time, space, and color) without external power sources or complex equipment. They simulate human trichromatic vision, converting infrared spectra into visible primary colors, thereby expanding the boundaries of human vision. This technology holds promise for applications in night vision, color blindness treatment, information encryption, and infrared spectral detection. Harvard's Michael H. Do praised the innovation in Cell Biomaterials, and Nature highlighted its potential to enhance our understanding of the world.

**Figure 2:**